# Mortality in female bodybuilding athletes

**DOI:** 10.1093/eurheartj/ehaf789

**Published:** 2025-10-20

**Authors:** Marco Vecchiato, Andrea Ermolao, Lara Zane, Silvia Giagio, Andrea Aghi, Stefano Palermi, Nicola Borasio, Alessandro Zorzi, Daniel Neunhaeuserer

**Affiliations:** Sports and Exercise Medicine Division, Department of Medicine, University of Padova, Via Giustiniani 2, Padova 35128, Italy; Sports and Exercise Medicine Division, Department of Medicine, University of Padova, Via Giustiniani 2, Padova 35128, Italy; Sports and Exercise Medicine Division, Department of Medicine, University of Padova, Via Giustiniani 2, Padova 35128, Italy; Department of Biomedical and Neuromotor Sciences (DIBINEM), Alma Mater Studiorum University of Bologna, Bologna, Italy; Sports and Exercise Medicine Division, Department of Medicine, University of Padova, Via Giustiniani 2, Padova 35128, Italy; Fisioterapia Osteopatia Raimondi di Giovanni e Daniele, Selvazzano Dentro, Padova, Italy; Department of Medicine and Surgery, UniCamillus-Saint Camillus International University of Health Sciences, Rome, Italy; Sports and Exercise Medicine Division, Department of Medicine, University of Padova, Via Giustiniani 2, Padova 35128, Italy; Institute of Mountain Emergency Medicine, Eurac Research, Bolzano, Italy; Department of Cardio-Thoraco-Vascular Sciences and Public Health, University of Padova, Padova, Italy; Sports and Exercise Medicine Division, Department of Medicine, University of Padova, Via Giustiniani 2, Padova 35128, Italy

**Keywords:** Bodybuilder, Sudden death, Sudden cardiac death, Doping, Anabolic steroids, Prevention

## Introduction

Female participation in competitive bodybuilding has steadily increased in recent decades.^1^ While the risks of mortality and sudden cardiac death (SCD) in male bodybuilders have recently been highlighted by our group,^2^ data on female athletes remain currently absent. This study aimed to address this gap by providing the first systematic evaluation of mortality rates in a large cohort of female bodybuilders during a long follow-up period. A secondary aim was to compare these findings with previously reported data of male athletes.

## Methods

The methodology mirrors the previously published analysis on male bodybuilders, covering the same 2005–2020 observation period, and applying identical data collection strategies.^2^ The only methodological differences concern the inclusion of three specific divisions, defined as Women’s Bodybuilding, Women’s Physique, and Figure categories, as well as the age cut-off to define master athletes, which is 35 years for women. Mortality rates were expressed per 100 000 athlete-years (AY), with 95% confidence intervals (CI) based on Poisson distribution. To compare mortality and SCD between sexes, incidence rate ratios (IRR) have been calculated, using female rates as reference and by providing 95% CI applying the exact Poisson method, without any adjustment for other covariates.

## Results

**Figure 1:**
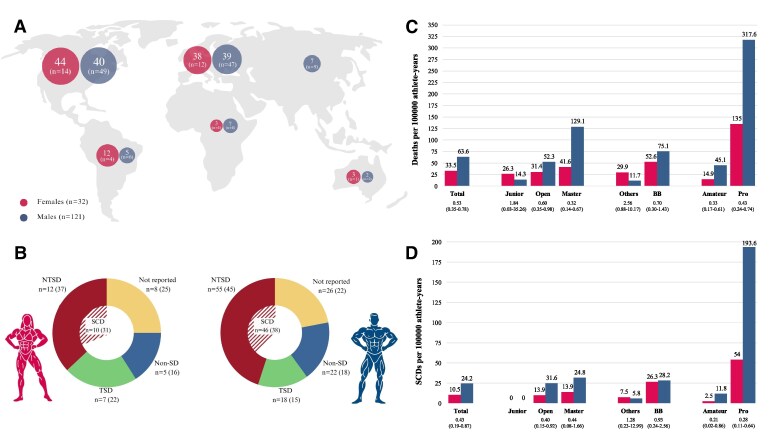
Overview of mortality data in female and male bodybuilding athletes. (*A*) Illustrates the geographical distribution of identified deaths, expressed as percentages and absolute numbers, stratified by sex. (*B*) Presents the distribution of causes of death in both sexes (percentages in brackets): non-sudden death, traumatic sudden death, non-traumatic sudden death, and unclassified cases. (*C* and *D*) Display the incidence rates per 100 000 athlete-years of all-cause mortality and sudden cardiac death, respectively, in the overall sample and across subgroups (e.g. junior, open, master, division type, amateur, and professional). ‘Others’ refers to Classic Physique division for men and Women’s Physique and Figure divisions for women, while ‘BB’ refers to Men’s and Women’s Bodybuilding. Incidence rate ratios with 95% confidence intervals comparing females to males are reported below each corresponding subgroup in both panels. NTSD, non-traumatic sudden death; SCD, sudden cardiac death; SD, sudden death; TSD, traumatic sudden death

Over a 16-year period, a total of 9447 unique female athletes participated in 700 International Fitness and Bodybuilding Federation events. Thirty-two deaths were recorded (mean age: 42.7 ± 9.8 years); most deceased athletes were from North America (*n* = 14, 44%) (*Figure 1A*).

A specific cause of death could be determined in 24 cases (75%). Of these, five were classified as non-sudden deaths (SD), while 19 were categorized as SD; seven traumatic and 12 non-traumatic SD. Ten SCDs have been identified (31% of total deaths) at an average age of 42.2 ± 10.9 years. Autopsy reports were available for only two professional athletes. One case showed no apparent cardiac abnormalities, while the other revealed histopathological findings consistent with myocarditis. Moreover, toxicological analyses of both athletes showed performance-enhancing drugs (PED) abuse, which could be documented in at least two additional deceased athletes.

The overall incidence rates of death and SCD in our sample were 33.51 (95% CI 22.92–47.30) and 10.47 (95% CI 5.02–19.26) per 100 000 AY, respectively. Only one athlete from the junior category was reported deceased, revealing a traumatic cause of death. There were nine deaths among master athletes with an average age of 51.6 ± 5.8 years, three of them classified as SCD. Women’s Bodybuilding was the division with the highest incidence rate of SCD, which was also notably higher among professional athletes, with 53.98 (95% CI 23.31–106.36) per 100 000 AY compared to 2.48 (95% CI .30–8.95) per 100 000 AY in amateurs.

Seven women died as currently competing athletes, considering a temporal range within 1 year after the last participation in a competition. Two SCDs have been registered, both occurred in close temporal proximity to a competitive event. The incidence rates of death and SCD in competing athletes were 40.87 (95% CI 16.43–84.22) and 11.68 (95% CI 1.41–42.19) per 100 000 AY, respectively.

Compared with our previous findings in male athletes, the causes of death mirrored those observed in males with SCD as the most represented (*Figure 1B*). When comparing incidence rates between sexes, the IRR for all-cause mortality was .53 (95% CI .35–.78) and for SCD .43 (95% CI .19–.87), indicating lower incidence rates in women (*Figure 1C* and *D*). Moreover, the IRR for SCD in professional athletes was .28 (95% CI .11–.64), highlighting a substantially reduced incidence rate in professional female compared to male bodybuilders.

## Discussion

This is the first study to quantify mortality and SCD incidence rates in a large cohort of female bodybuilding athletes. SCD appeared to be the most common cause of death, in line with findings in male athletes.^2^ However, since the cause of death could not be determined in 25% of cases, this conclusion should be interpreted with caution. Interestingly, none of the two available autopsy reports revealed cardiomegaly or severe left ventricular hypertrophy, findings that contrast with the structural cardiac abnormalities observed in deceased male bodybuilders.^3^ This discrepancy may suggest sex-specific differences in cardiac remodelling.^4^ Indeed, generally the incidence of SCD in female athletes is typically lower when compared with males, also associated with significant sex-related adaptations.^5,6^ Nevertheless, it is noteworthy that many of the same risk determinants related to mortality in male bodybuilding athletes, such as extreme strength training, drastic weight manipulation strategies and PED abuse, are likely to be present in female athletes as well.^7^ Although existing literature suggests a lower prevalence of PED utilization among female athletes compared to males,^8^ the impact of such substance abuse may still be clinically relevant in this population.

Furthermore, deaths due to suicide and homicide accounted for nearly 13% of all cases in the female cohort, more than four times the proportion observed in male bodybuilders. This difference may reflect greater psychosocial vulnerability among female athletes, potentially influenced by gender-specific stressors, dynamics which have been previously associated with increased psychological strain among female bodybuilders.^9^ These findings highlight the urgent need for integrated support strategies, including mental health screening, education, and gender-sensitive prevention frameworks in competitive bodybuilding.^10^

When comparing these study results with similar analyses in other sports, the observed SCD incidence rate in female bodybuilders significantly exceeds that reported in other female athletic populations.^11^ Nevertheless, it remains challenging to offer a fair comparison with other studies due to the unique characteristics of this specific athletic population. Additionally, the present study carries certain methodological limitations, as previously discussed, which should be considered when interpreting the findings: retrospective design, reliance on publicly available data, the absence of adjustment for potential confounders due to limited available information, as well as a lack of systematic autopsy and toxicological reports may lead to underreporting or misclassification of deaths.^2^ Given the increased mortality risk observed in female bodybuilding athletes, there is a pressing need for targeted preventive strategies, including structured medical evaluations, educational initiatives and tighter monitoring of PED abuse, also to promote a cultural shift in this sport discipline.

## Conclusion

While the mortality rates in female bodybuilders were lower than in men, these findings support the hypothesis that the health risks associated with bodybuilding are not limited to male athletes. Extreme training, eating/fasting behaviour, dehydration strategies, and uncontrolled PED abuse, may contribute to elevated mortality risk also in women, particularly at a professional level.

## Declarations

### Data Availability

Complete dataset can be shared after reasonable request to corresponding author.

### Pre-registered Clinical Trial Number

None supplied.
